# Type V Collagen as a Critical Regulator of Fibrillar Matrix Remodeling in a Murine Model of Systemic Sclerosis

**DOI:** 10.3390/cells14231865

**Published:** 2025-11-26

**Authors:** Zelita Aparecida J. Queiroz, Ana Paula P. Velosa, Vitória Elias Contini, Juliana Sampaio-Silva, Sergio Catanozi, Antonio dos Santos Filho, Solange Carrasco, Thays de Matos Lobo, Lizandre Keren R. da Silveira, Fabíola Santos Zambon Robertoni, Camila Machado Baldavira, Sandra M. Fernezliam, Aritania S. Santos, Cláudia Goldenstein-Schainberg, Percival Degrava Sampaio-Barros, Débora Levy, Vera Luiza Capelozzi, Walcy Rosolia Teodoro

**Affiliations:** 1 Divisao de Reumatologia, Faculdade de Medicina FMUSP, Universidade de Sao Paulo, Sao Paulo 01246-903, SP, Brazil; zelitaaparecida@gmail.com (Z.A.J.Q.); veliascontini@gmail.com (V.E.C.); antoniodossantosfilho1965@gmail.com (A.d.S.F.); socarrasco2@gmail.com (S.C.); thaysmatos@usp.br (T.d.M.L.); lizandre_keren@usp.br (L.K.R.d.S.); fabiola.robertoni@alumni.usp.br (F.S.Z.R.); 2 Divisao de Reumatologia, Hospital das Clinicas HCFMUSP, Faculdade de Medicina, Universidade de Sao Paulo, Sao Paulo 05403-010, SP, Brazil; apvelosa@gmail.com (A.P.P.V.); claudia.schainberg@hc.fm.usp.br (C.G.-S.); pdsampaiobarros@uol.com.br (P.D.S.-B.); 3 Lipids, Oxidation, and Cell Biology Group, Laboratory of Immunology (LIM19), Heart Institute (InCor), Hospital das Clínicas, Faculdade de Medicina, Universidade de São Paulo (HCFMUSP), São Paulo 05403-900, SP, Brazil; jukisbio@gmail.com (J.S.-S.); d.levy@hc.fm.usp.br (D.L.); 4 Laboratorio de Lipides (LIM-10), Hospital das Clinicas HCFMUSP, Faculdade de Medicina, Universidade de Sao Paulo, Sao Paulo 01246-903, SP, Brazil; catanozi@usp.br; 5 Laboratory of Lung Histomorphometry and Genomics, Department of Pathology, Faculdade de Medicina FMUSP, Universidade de Sao Paulo, Sao Paulo 01246-903, SP, Brazil; ca.mbaldavira@gmail.com (C.M.B.); sandra.f@fm.usp.br (S.M.F.); vera.capelozzi@fm.usp.br (V.L.C.); 6 Laboratorio de Carboidratos e Radioimunoensaios (LIM18), Faculdade de Medicina FMUSP, Universidade de Sao Paulo, Sao Paulo 01246-903, SP, Brazil; aritania@alumni.usp.br

**Keywords:** collagen V, systemic sclerosis, skin, fibroblast, experimental model, fibrillogenesis

## Abstract

Type V collagen (Col V) has been implicated in the development of fibrosis in systemic sclerosis (SSc). In this study, we aimed to investigate the role of Col V in fibrillar matrix remodeling and fibroblast differentiation using an experimental SSc model. Skin fibroblasts from healthy C57BL/6 mice were stimulated in vitro with 25 and 50 μg of Col V to assess fibrillar collagen expression. An SSc model was induced in C57BL/6 mice by immunization with Col V emulsified in Freund’s adjuvant (IMU-COLV), with animals assigned to 15-, 30-, and 45-day IMU-COLV or control groups. In vitro, Col V stimulation caused a dose-dependent increase in myofibroblast markers (α-SMA, Col I, and Col V) and altered fibrillar collagen structure. Immunofluorescence revealed thickened Col V and Col III fibrils around myofibroblasts and the formation of a spiderweb-like matrix. In vivo, fibrosis progressed over time, characterized by increased myofibroblast accumulation and elevated Col I and Col V levels. Histological analysis revealed fibrillar disorganization and aggregated collagen fibers resembling early-stage human SSc. These findings suggest that enhanced Col V synthesis disrupts the fibrillar matrix, promoting myofibroblast differentiation and collagen deposition, which are hallmarks of SSc-related fibrosis.

## 1. Introduction

Systemic sclerosis (SSc) is an autoimmune rheumatic disease characterized by endothelial cell injury, sustained myofibroblast activation, and excessive extracellular matrix (ECM) secretion, leading to progressive fibrosis of the skin and internal organs [1,2,3]. Because many of these features are resistant to current therapies, SSc has a higher mortality rate than other rheumatic diseases [4,5]. Despite extensive research, the mechanisms driving fibrosis, persistent autoimmunity, and vasculopathy remain incompletely understood. Further investigation is needed to clarify how the matrix microenvironment, particularly altered collagen composition, influences cellular responses such as fibroblast signaling and fibrillogenesis. These insights may help identify biomarkers and risk factors, support early diagnosis, improve treatment outcomes, and enhance prognostication.

Collagen is a diverse family of structural ECM proteins that provide mechanical support and regulate cell behavior in connective tissues. Fibrillar collagens, including types I, III, and V, are major components of skin and internal organs, forming heterotypic fibrils that determine tissue tensile strength and elasticity. Dysregulation of these collagens is central to the pathogenesis of several rheumatologic diseases, such as SSc, rheumatoid arthritis, and lupus, where altered collagen deposition contributes to fibrosis, chronic inflammation, and impaired tissue function [6,7,8]. In SSc, progressive fibrosis is associated with overproduction of type I collagen (Col I) and type III collagen (Col III), which drive skin thickening and loss of elasticity [9,10].

Emerging evidence indicates that type V collagen (Col V), a minor fibrillar collagen that co-assembles with Col I and III, is also altered early in SSc. Col V plays a critical role in regulating fibril nucleation and diameter, and its abnormal expression can disrupt the structural organization of Col I/III/V fibrils [11,12]. Importantly, Col V may act as a sequestered autoantigen, becoming exposed to the immune system during tissue injury and contributing to autoimmune responses in multiple pathological conditions [13,14,15,16]. In SSc, elevated expression of *COL5A1* and *COL5A2* correlates with disease activity and skin thickening [9,17].

We previously identified abnormal Col V expression in the skin of patients with SSc, which correlated with disease severity [17]. In lung biopsies and fibroblasts from patients with SSc, we also observed increased Col V gene expression and abnormal Col V synthesis, both associated with interstitial fibrosis and impaired lung function [18,19]. Experimental models further support the pathogenic role of Col V: Teodoro and colleagues developed an experimental SSc model using Col V immunization in C57BL/6 mice, reporting histological, immunological, and vascular alterations that closely mimic human SSc [20]. Their findings suggest that deposition of Col V/anti-Col V immune complexes on the vascular surface triggers endothelial injury, exposing previously hidden Col V beneath the basement membrane, which then acts as a neoantigen and initiates inflammation, driving autoimmunity [21].

Although the involvement of Col V in fibrosis is well-documented, the precise mechanisms by which it contributes to SSc remain unclear. Alterations in Col V likely affect collagen fibril structure, fibroblast signaling, and ECM remodeling, yet the specific pathways mediating these effects have not been fully explored. We hypothesized that early structural changes in Col V, triggered by tissue injury, activate matrix proteins and modulate signaling pathways, promoting fibroblast differentiation into myofibroblasts. This process would significantly affect collagen production and contribute to the fibrotic features characteristic of SSc. We further propose that microenvironments enriched with Col V may activate additional signaling pathways, leading to changes in cell phenotype and gene expression.

To test these hypotheses, we aimed to develop a novel cellular model to examine the interaction between Col V and fibroblast behavior in vitro and investigated whether the structural changes in Col V observed in early experimental SSc are sufficient to trigger fibroblast activation and collagen dysregulation. This research provides critical insights into the role of Col V in fibrosis and its potential as a therapeutic target in SSc while also contributing to a deeper understanding of matrix-related signaling processes that drive disease progression.

## 2. Materials and Methods

### 2.1. Animals and Study Design

Animals were randomized to experimental groups using a computer-generated random sequence (Excel RAND()/R sample()), prepared by an investigator not involved in animal handling. Group allocation was implemented by assigning each animal a study ID and then allocating IDs to groups according to the random list. No allocation concealment was used; the person performing the allocation was independent from the investigator performing outcome assessment to reduce bias. Female C57BL/6 mice were used in all experiments. We used female mice exclusively for this study because biological rationale: for example, the disease has a possible sex-specific incidence, in which primarily affects women.

Sample size was determined a priori using power calculations for the primary outcome (effect: difference in mean collagen area fraction between treatment and control). Using a two-sample *t*-test, two-sided α = 0.05 and power (1 − β) = 0.80, an effect size (Cohen’s d) of d was assumed based on pilot data/previous publications [20].

Fifty-four female C57BL/6 mice (6–7 weeks old) were obtained from the institutional animal facility and housed under specific pathogen-free conditions. Mice had free access to food and water and were maintained in a temperature-controlled room (22–24 °C) with a 12 h light/dark cycle. All procedures were conducted in accordance with the guidelines approved by the Committee on Ethical Use of Laboratory Animals, Faculty of Medicine, University of São Paulo (protocol numbers 1416/2019 and 1543/2020), ensuring compliance with ethical standards for animal welfare. The study also followed relevant guidelines, including the Animal Research: Reporting of In Vivo Experiments (ARRIVE) guidelines and international regulations.

To investigate early-stage skin involvement in SSc, mice were divided into an experimental group immunized with human placental Col V (IMU-COLV, *n* = 30) and a control group (CT, *n* = 24). The IMU-COLV group was further subdivided according to time of euthanasia (*n* = 10 per time point: 15, 30, or 45 days postimmunization) to assess temporal changes in skin fibrosis and fibroblast activation. All subsequent analyses, including histological, immunohistochemical, and cellular experiments, were performed using tissues from each individual mouse, with the number of biological replicates indicated in the figure legends.

### 2.2. Col V Immunization Protocol

Mice were randomly assigned to either control (CT) or Col V-immunized (IMU-COLV) groups using a computer-generated randomization list. Group sizes (8–10 mice per group) were based on preliminary studies and published data, providing >80% power to detect biologically meaningful differences in collagen deposition, α-SMA expression, and other primary outcomes. All animals were maintained under standard laboratory conditions with ad libitum access to food and water.

**Figure 1:**
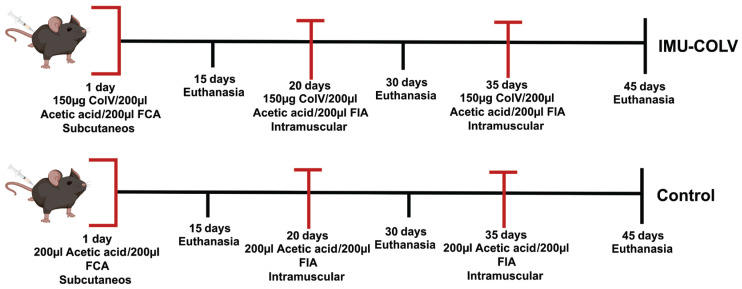
Experimental design. The diagram illustrates the immunization protocol with Col V in C57BL/6 mice, detailing the dose, the period of antigen inoculation, and euthanasia of the animals. IMU-COLV: immunized with Col V. FCA: Freund’s complete adjuvant; FIA: Freund’s incomplete adjuvant. Mice created with BioRender.com.

On day 1, mice in the IMU-COLV group were immunized subcutaneously with 150 µg of human placental Col V (Sigma–Aldrich, St. Louis, MO, USA) diluted in 10 mM acetic acid and emulsified in an equal volume of Freund’s complete adjuvant (Sigma–Aldrich) to enhance the immune response. On day 20, a booster dose of Col V (150 µg/200 µL) was administered subcutaneously using incomplete Freund’s adjuvant (Sigma–Aldrich), followed by a third booster dose (150 µg/200 µL Col V in incomplete Freund’s adjuvant) 15 days later (Figure 1). The control group (CT, *n* = 24) received the same adjuvant regimen without Col V. For preventive analgesia, tramadol hydrochloride (40 mg/kg body weight) was administered subcutaneously 1 h before immunization and every 8 h for 48 h after the procedure.

At 15, 30, or 45 days postimmunization, animals were euthanized by intraperitoneal injection of an anesthetic overdose (ketamine hydrochloride, 300 mg/kg body weight, and xylazine hydrochloride, 30 mg/kg body weight), followed by cervical dislocation. For each experimental group and time point, *n* = 10 mice from the IMU-COLV group and *n* = 8 mice from the CT group were euthanized. Skin samples were collected immediately and processed for histological analysis and molecular profiling. All samples were analyzed individually, and the number of biological replicates is indicated in the figure legends.

This study design allowed assessment of early alterations in collagen composition and fibroblast activation at different stages of disease progression.

No animals or samples were excluded post hoc; all collected data are reported in the figures and supplementary tables.

### 2.3. In Vitro Fibroblast Culture and Stimulation

Skin fibroblasts were cultured from skin biopsies obtained from healthy or Col V–immunized C57BL/6 mice. Skin samples were disinfected with 70% ethanol, transferred to a sterile Petri dish, and cut into fragments of approximately 1 mm^2^. Fibroblasts were isolated using the explant method. Cells were seeded on scaffolds and stimulated with Col V at 25 μg/mL or 50 μg/mL for 24 or 48 h. Cultures were maintained in Dulbecco’s Modified Eagle’s Medium (DMEM; Gibco, Grand Island, NY, USA) supplemented with 10% fetal bovine serum, 100 U/mL penicillin (Sigma–Aldrich), and 100 μg/mL streptomycin (Sigma–Aldrich) in a humidified 5% CO2 incubator at 37 °C. The medium was replaced every 72 h, and cells were passaged at 80% confluence. Enzymatic dissociation was performed using 0.05% trypsin-ethylenediaminetetraacetic acid (EDTA; Sigma-Aldrich), and cells were subcultured in 75 cm^2^ tissue culture flasks. Fibroblasts at passage 3 (P3) were used for further experiments.

Fibroblast attachment, myofibroblast differentiation (α-SMA), and ECM production (Col I, Col III, Col V) were assessed using immunofluorescence imaging. Two samples were collected in triplicate, and nine regions from each sample were analyzed.

### 2.4. Hematoxylin–Eosin and Masson’s Trichrome Staining

Mouse skin tissues were fixed in 10% formaldehyde (Sigma-Aldrich) for 24 h at 4 °C and embedded in paraffin. Five-micrometer-thick sections were cut using a microtome, and at least three nonconsecutive sections per mouse were processed for staining. Sections were deparaffinized in xylene, rehydrated through a graded ethanol series (100%, 95%, and 70%), and washed in phosphate-buffered saline. For hematoxylin and eosin (H&E) staining, sections were stained with hematoxylin for 5 min, rinsed under running tap water, and counterstained with eosin for 2 min. The sections were then dehydrated through a graded ethanol series, cleared in xylene, and mounted with coverslips using Permount^®^ mounting medium (Fisher Scientific GmbH, Schwerte, Germany).

For Masson’s Trichrome staining, sections were sequentially stained with Weigert’s iron hematoxylin working solution (Sigma-Aldrich), Biebrich scarlet-acid fuchsin solution, and aniline blue solution, following standard protocols. Briefly, the sections were stained in Weigert’s iron hematoxylin for 10 min, rinsed with distilled water, stained in Biebrich scarlet-acid fuchsin solution for 5 min, and rinsed again. They were subsequently stained in aniline blue for 10 min. Between each step, sections were washed in distilled water to remove excess dye and prevent background staining. After staining, the sections were dehydrated through graded ethanol, cleared in xylene, and mounted with coverslips. For each experimental group, skin samples from all mice (IMU-COLV, *n* = 10; CT, *n* = 8) were analyzed. Histological evaluation was performed using three sections per mouse for all mice in each group and time point.

The stained sections were examined and imaged under a light microscope (Olympus BX-51, Olympus Co., Tokyo, Japan). Histological features, including collagen deposition and skin architecture, were assessed in a blinded manner to quantify fibrosis in experimental SSc.

Fibrillar organization and matrix morphology were evaluated qualitatively by two independent observers blinded to the experimental groups. Descriptive terms such as ‘spiderweb-like matrix’ and ‘fibrillar disorganization’ refer to reproducible histological patterns characterized by irregular collagen orientation, loss of parallel fiber alignment, and disrupted connective network structure

### 2.5. Immunofluorescence

For cell culture-based immunofluorescence, skin fibroblasts from healthy animals were seeded in a black 96-well plate at a density of 5 × 10^4^ cells/cm^2^. Once cells reached 80–90% confluence, they were treated with different concentrations of Col V (25 and 100 μg/mL) for 24 or 48 h. Following treatment, cells were fixed in 4% paraformaldehyde for 2 h at 4 °C, permeabilized with 0.1% Triton X-100 for 15 min, and blocked with 5% bovine serum albumin. Cells were then incubated overnight at 4 °C with the following primary antibodies: rabbit anti-Col III polyclonal antibody (1:200; Rockland Immunochemicals, Pottstown, PA, USA), rabbit anti-Col V polyclonal antibody (1:100; Rockland Immunochemicals), goat anti-Col I polyclonal antibody (1:1500 US Biological, Salem, MA, USA), or mouse monoclonal anti-alpha-smooth muscle actin (α-SMA) antibody (1:100; Santa Cruz Biotechnology, Dallas, TX, USA). After washing, cells were incubated for 2 h at room temperature with one of the following secondary antibodies: Alexa Fluor 488–conjugated rabbit anti-immunoglobulin G (IgG) (1:500; Invitrogen Life Technologies Co., Carlsbad, CA, USA), Alexa Fluor 546–conjugated goat anti-IgG (1:500; Invitrogen Life Technologies), or Alexa Fluor 488–conjugated mouse anti-IgG (1:500; Invitrogen, Life Technologies). Nuclei were counterstained with Hoechst 33342. Fluorescence intensity and the percentage of positive cells were quantified using the ImageXpress Micro High Content System and Cell Scoring MetaXpress software, version 5 (Molecular Devices, San Jose, CA, USA).

For immunofluorescence on fibroblasts from Col V-immunized mice, cells were seeded on coverslips, fixed, permeabilized, and blocked as described above. The cells were incubated overnight with the following primary antibodies, diluted in phosphate-buffered saline: rabbit anti-Col I produced in the laboratory (1:100) [22], anti-Col III (1:200), anti-Col V (1:100), anti-transforming growth factor-β (TGF-β; 1:50; Santa Cruz Biotechnology, Dallas, TX, USA), anti-signal transducer and activator of transcription 3 (STAT3; 1:100; Santa Cruz Biotechnology), mouse monoclonal anti-α-SMA (1:100), or anti-integrin α5/CD49e (1:100; Novus Biologicals, Centennial, CO, USA). The cells were then incubated with the corresponding Alexa Fluor 488–conjugated anti-rabbit IgG or Alexa Fluor 488–conjugated mouse anti-IgG antibodies (1:200) prepared in Evans blue. Nuclear staining was performed with 4′,6-diamidino-2-phenylindole (DAPI; Abcam, Cambridge, UK) for 5 min. Finally, the cells were mounted and imaged using a fluorescence microscope (Olympus BX-51).

For immunofluorescence of skin tissue, 4 µm skin sections were mounted on slides, subjected to antigen retrieval using bovine pepsin, and blocked with 5% bovine serum albumin to prevent nonspecific binding. The slides were incubated overnight at 4 °C with primary antibodies, including rabbit polyclonal anti-Col I (1:50; Rockland Immunochemicals, Pottstown, PA, USA), anti-Col III (1:50; Rockland Immunochemicals), or anti-Col V produced in the laboratory (1:800) [22]. After washing, the slides were incubated with Alexa Fluor 488–conjugated anti-rabbit IgG secondary antibody, mounted with glycerol, and imaged using a fluorescence microscope (Olympus BX-51, Olympus Corp., Tokyo, Japan).

For colocalization assays, sections were incubated with goat anti-Col I polyclonal antibody (1:1500), rabbit anti-Col V polyclonal antibody (1:300), and mouse anti-α-SMA monoclonal antibody (1:30), followed by Alexa Fluor 488 (green)-conjugated anti-rabbit IgG and Alexa Fluor 546 (red)-conjugated anti-mouse and anti-rabbit IgG secondary antibodies. Nuclei were counterstained with DAPI, and images were acquired using the Olympus BX-51 fluorescence microscope.

Negative controls (secondary antibody only) were included for all antibodies and are provided in Figures S1 and S2.

### 2.6. Immunohistochemistry

Skin biopsy sections (3–4 µm) were deparaffinized and treated with 0.3% hydrogen peroxide for 5 min to block endogenous peroxidase activity. Antigen retrieval was performed using bovine pepsin (4 mg/mL in 0.01 N glycine buffer) for 30 min at 37 °C for α-SMA, whereas vascular endothelial growth factor (VEGF) was subjected to heat-induced antigen retrieval using citrate buffer (pH 6.0) at 125 °C for 1 min in a pressure cooker. The sections were then incubated overnight at 4 °C with primary antibodies: mouse monoclonal anti-α-SMA (1:50) and anti-VEGF (1:400; Santa Cruz Biotechnology). Detection was carried out using a biotin-streptavidin-peroxidase system, and the signal developed with 3,3-diaminobenzidine as the chromogen. Sections were counterstained with Harris hematoxylin.

Negative controls (secondary antibody only) were included for all antibodies and are provided in Figure S3.

### 2.7. Digital Morphometry

Slides immunostained for Col I, III, and V were analyzed using Image-Pro Plus 6.0 (Media Cybernetics, Rockville, MD, USA). Images were acquired using fluorescence and light microscopy at standardized magnifications and identical acquisition parameters across all groups. Investigators were blinded to experimental group assignment during image acquisition and analysis. Quantitative measurements, including collagen fiber intensity, dermal thickness, and α-SMA+ cell counts, were performed using automated or semi-automated image analysis software. Briefly, ten fields from the papillary and reticular dermis were evaluated at 400× magnification. Collagen content in the selected fields was quantified as the percentage of collagen, calculated by dividing the green fluorescence area by the total tissue area. For α-SMA and VEGF analysis, six fields of skin tissue were examined at 1000× magnification. Positive cells were counted and expressed as a proportion of the total cells in each field [23].

### 2.8. Biochemistry

Total skin collagen deposition was measured by determining 4-hydroxyproline content, with modifications to a previously described method [24]. Skin samples were freeze-dried, weighed, and hydrolyzed in 6 N HCl at 110 °C for 22 h. Hydroxyproline was quantified spectrophotometrically at 560 nm, and results were expressed as nanograms of 4-hydroxyproline per milligram of protein [24]. For each experimental group and time point, skin samples from all mice were analyzed (IMU-COLV, *n* = 8; CT, *n* = 7).

### 2.9. Quantitative Reverse Transcription Polymerase Chain Reaction

Total RNA was isolated using the standard Trizol^®^ protocol (Invitrogen Life Technologies). Expression of collagen type III α1 chain (*Col3a1*) and collagen type V α1 chain (*Col5a1*) was assessed by quantitative reverse transcription polymerase chain reaction (RT-qPCR). RNA was reverse-transcribed into complementary DNA (cDNA) using the High-Capacity cDNA Reverse Transcription Kit (Applied Biosystems, Foster City, CA, USA). RT-qPCR reactions were prepared with the Platinum SYBR Green qPCR SuperMix-UDG Kit (Invitrogen Life Technologies). cDNA synthesis and amplification were performed on a StepOne thermal cycler (Applied Biosystems) using 1000 ng of total RNA per sample. Relative expression was calculated using the 2^−ΔΔCT^ method, with β-2-microglobulin as the reference gene. The primer sequences used were as follows: *Col3a1*—Sense 3′–5′: GCA CAG CAG TCC AAC GTA GA; Antisense 5′–3′: TCT CCA AAT GGG ATC TCT GG. *Col5a1*—Sense 3′–5′: GGT CCC TGA CAC ACC TCA GT; Antisense 5′–3′: TGC TCC TCA GGA ACC TCT GT. *B2m*—Sense 3′–5′: CAT GGC TCG CTC GGT GAC C; Antisense 5′–3′: AAT GTG AGG CGG GTG GAA CTG. For each experimental group, skin samples from all mice were analyzed (IMU-COLV, *n* = 7; CT, *n* = 5).

### 2.10. Statistical Analysis

Data distribution was assessed to determine the use of parametric or nonparametric tests using GraphPad Prism version 8.0.2 (GraphPad Software, San Diego, CA, USA). One-way analysis of variance (ANOVA) was used to compare group means, with Tukey’s or Sidak’s test for post hoc analysis of normally distributed data. For non-normally distributed data, the Kruskal–Wallis test followed by Dunn’s test for pairwise comparisons was applied.

Effect size estimates (η^2^ for ANOVA and ε^2^ for Kruskal–Wallis) and their 95% confidence intervals were computed in RStudio version 4.4.1 (R Foundation for Statistical Computing, Vienna, Austria) using the effectsize package. The magnitude of the effects was interpreted according to conventional thresholds (small, medium, large). In analyses with very small sample sizes where reliable effect size estimation was not feasible, only descriptive statistics and *p*-values are reported (Tables S1 and S2).

## 3. Results

### 3.1. Time- and Concentration-Dependent Effects of Collagen V on Myofibroblast Differentiation and Collagen Synthesis in Mouse Fibroblasts

**Figure 2:**
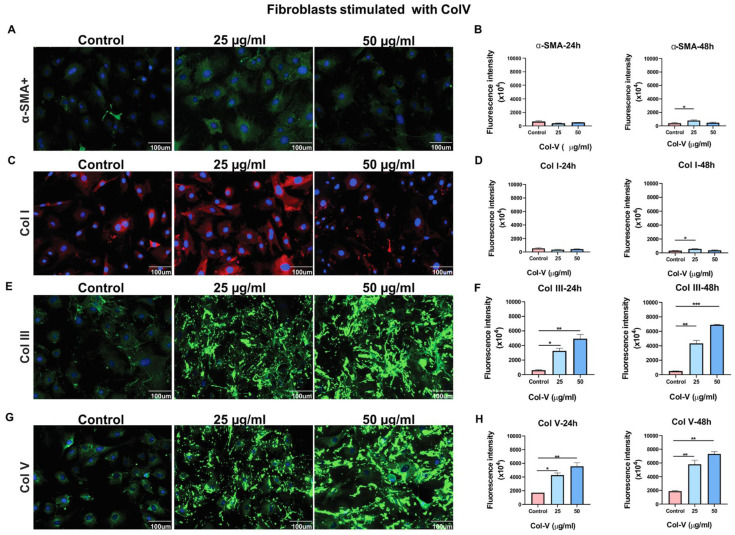
Representative images at 48 h. Expression of Col I, Col III, Col V, and α-SMA in skin fibroblasts from healthy mice stimulated with Col V. (**A**) Immunofluorescence (green) shows α-SMA expression in the cytoplasm of control fibroblasts and fibroblasts stimulated with Col V. (**B**) Graphical representation of the amount of α-SMA after 24 and 48 h of incubation with Col V. (**C**) Immunofluorescence (red) shows Col I expression in the cytoplasm of fibroblasts stimulated with Col V. (**D**) Graphical representation of the amount of Col I after 24 and 48 h of incubation with Col V. (**E**) Immunofluorescence (green) shows Col III expression in the cytoplasm of fibroblasts stimulated with Col V. (**F**) Graphical representation of the amount of Col III after 24 and 48 h of incubation with Col V. (**G**) Immunofluorescence (green) shows the Col V expression in the cytoplasm of fibroblasts stimulated with Col V. (**H**) Graphical representation of the amount of Col V after 24 and 48 h of incubation with Col V. GraphPad Prism version 8; one-way ANOVA, followed by Tukey’s post hoc test. (original magnification: 400×). Significance levels: *p* < 0.05 (*), *p* < 0.01 (**), *p* < 0.001 (***).

After 24 h of Col V stimulation, no significant increase in α-SMA^+^ fibroblasts (myofibroblasts) was observed compared with CT (Figure 2A,B). However, after 48 h, a significant increase was detected at 25 μg/mL (*p* = 0.0316; Table S1), indicating a delayed response to Col V with increased myofibroblast differentiation.

Col I synthesis increased significantly at 25 μg/mL (*p* = 0.0319) (Figure 2C,D), correlating with myofibroblast differentiation and suggesting active ECM remodeling. Col III, a marker of early fibrosis, was significantly elevated at both 24 h (25 μg/mL, *p* = 0.0125; 50 μg/mL, *p* = 0.0030) and 48 h (25 μg/mL, *p* = 0.0012; 50 μg/mL, *p* < 0.001) (Figure 2E,F), indicating both early and sustained deposition. Col V expression also increased significantly at 24 h (25 μg/mL, *p* = 0.0116; 50 μg/mL, *p* = 0.0036) and 48 h (25 μg/mL, *p* = 0.0047; 50 μg/mL, *p* = 0.0018) (Figure 2G,H; Table S1), suggesting a self-reinforcing accumulation in the ECM that could amplify fibrotic responses through a positive feedback loop.

At 48 h, both single and clustered fibroblasts attached to the scaffold fibers. In Col V–immunized mice, histological evaluation demonstrated a disorganized fibrillar matrix composed of irregularly oriented and intersecting collagen fibers that produced a characteristic spiderweb-like pattern. Collagens III and V were distributed within this network, forming interlacing structures between fibers and highlighting marked remodeling of the fibrillar architecture (Figure 2E,G).

### 3.2. Histological Analysis of Collagen Remodeling and Myofibroblast Activity in Response to Collagen V Stimulation

**Figure 3:**
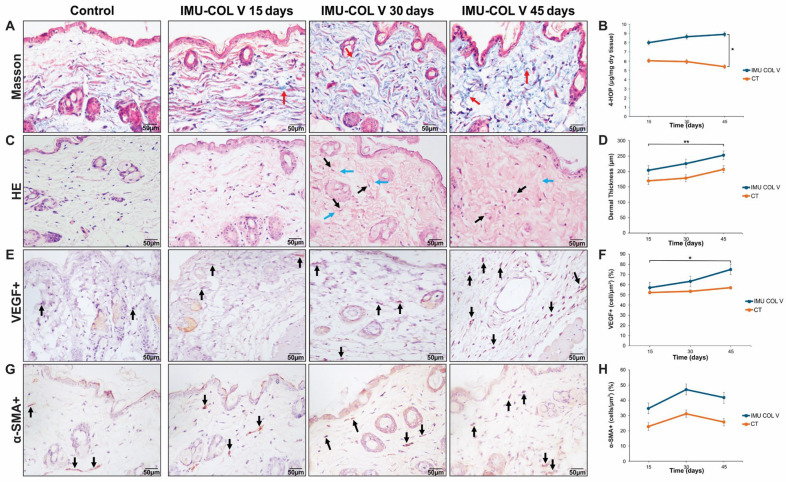
Changes in skin morphology, dermal thickness, total collagen, and immunohistochemical analysis of VEGF and α-SMA in C57BL/6 mice after immunization with Col V. (**A**) Staining with Masson’s trichrome. We identified the distribution of Col fibers in blue (red arrow). Note the intense disorganization and dissociation of the Col fibrils in the IMU-COLV group after 45 days. (**B**) Graph of 4-hydroxyproline showing the significant difference between the 45-day and CT groups. (**C**) Histological sections of skin tissue stained with H&E from the CT, 15-day, 30-day, and 45-day groups (arrow: myofibroblast; blue arrow: collagen fiber). (**D**) Graphical representation of the papillary dermis thickness values in the IMU-COLV and CT groups. (**E**) Positive cells are stained brown (arrows). We observed an increase in the number of VEGF^+^ cells in the 45-day group compared with the 15-day group. (**F**) Graphical representation of the amount of VEGF^+^. (**G**) We observed α-SMA^+^ positive cells in the dermal region in the groups (arrows). (**H**) Graphical representation of the amount of α-SMA^+^. GraphPad Prism version 8; one-way ANOVA followed by Sidak’s post hoc test and the Kruskal–Wallis test followed by Dunn’s post hoc test. (original magnification: 400×). Significance levels: *p* < 0.05 (*), *p* < 0.01 (**) (*n* = 8–10 per group).

Masson’s trichrome and immunohistochemical staining revealed progressive collagen deposition in the dermis of IMU-COLV mice over time. Collagen fibers were thin and lightly stained blue on day 15, becoming thicker and more intensely stained by day 30 (Figure 3A, red arrow), and reaching maximal deposition with spiral- or plate-like fiber structures by day 45 (Figure 3A,C). Spindle-shaped myofibroblasts appeared around day 30, with their number and associated fibers increasing by day 45 (Figure 3C, black arrows). Some collagen fibers fused, particularly around vessels and regions of active remodeling (blue arrow).

VEGF positivity was detected in small-vessel endothelial cells, with stronger expression at day 45 than day 15 (*p* = 0.0045) (Figure 3E,F), suggesting enhanced angiogenesis or vascular remodeling.

**Figure 4:**
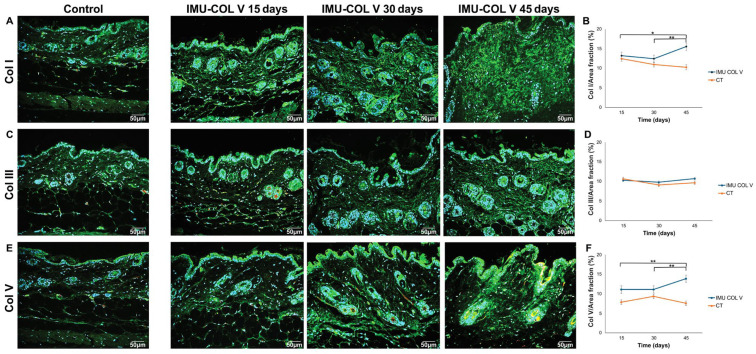
Changes in the number of Col fibers in the skin following Col V immunization of C57BL/6 mice. Immunofluorescence of Col I, Col III, and Col V. (**A**) Col I shows a pattern of thick fibers along the papillary dermis and reticular dermis in the skin of the IMU-COLV and CT groups. (**B**) Graphical representation of the amount of Col I. (**C**) CoI III shows a fine fibrillar pattern in the skin of the IMU-COLV and CT groups. (**D**) Graphical representation of the amount of Col III. (**E**) Col V shows a fine fibrillar pattern evident in the papillary and reticular dermis and around the appendages in the skin of the IMU-COLV groups. (**F**) Graphical representation of the amount of Col V. GraphPad Prism version 8; one-way ANOVA followed by Sidak’s post hoc test and the Kruskal–Wallis test followed by Dunn’s post hoc test. (original magnification: 400×). Significance levels: *p* < 0.05 (*), *p* < 0.01 (**) (*n* = 8–10 per group).

Progressive deposition of Col I, Col III, and Col V was observed. Col I intensity (green birefringence) increased from day 15 to 45, with significantly greater deposition at day 45 (*p* = 0.0393; *p* = 0.0016) (Figure 4A,B). Biochemical analysis using the 4-hydroxyproline assay confirmed a significant increase at day 45 in IMU-COLV mice compared with CT (*p* = 0.0038) (Figure 3B; Table S2).

**Figure 5:**
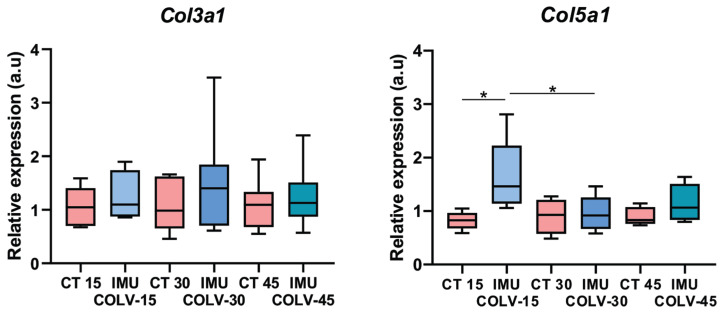
Relative expression of *Col3a1* and *Col5a1* genes in the skin of mice from the IMU-COLV and CT groups. Data are presented as mean ± standard error. Statistical analysis was performed using one-way ANOVA followed by Tukey’s post hoc test (GraphPad Prism version 8). Significance levels: *p* < 0.05 (*) (*n* = 5–7 per group).

Col III fibrils filled the dermis at days 15 and 30. By day 45, small fibrillar aggregates appeared in the papillary dermis and around thickened vessel walls (Figure 4C). These patterns were absent in CT mice, although quantitative differences were not statistically significant. *Col3a1* gene expression was similarly unchanged (Figure 5).

Col V fibers increased over time, appearing thin at day 15, more prominent at day 30, and aggregated with fibrillar disorganization by day 45, particularly around vessels and skin appendages (Figure 4E). Quantitative analysis confirmed significant increases between 15 and 45 days (*p* = 0.0020) and between the 30- and 45-day groups (*p* = 0.0026) (Figure 4F). *Col5a1* gene expression was significantly higher at day 15 compared with CT (*p* = 0.0155) and day 30 (*p* = 0.0369) (Figure 5).

α-SMA+ cells were present in the dermis and vascular walls at all time points, although quantitative analysis revealed no significant differences compared with CT (Figure 3G,H). Although α-SMA quantification did not reveal significant group differences, localized perivascular enrichment of α-SMA–positive cells suggest early myofibroblast differentiation. Similarly, the fine fibrillar collagen pattern observed may reflect initial extracellular matrix remodeling, preceding detectable transcriptional upregulation of *Col3a1*.

### 3.3. Colocalization of Collagen Fibers, Myofibroblasts, and Signaling Pathways

**Figure 6:**
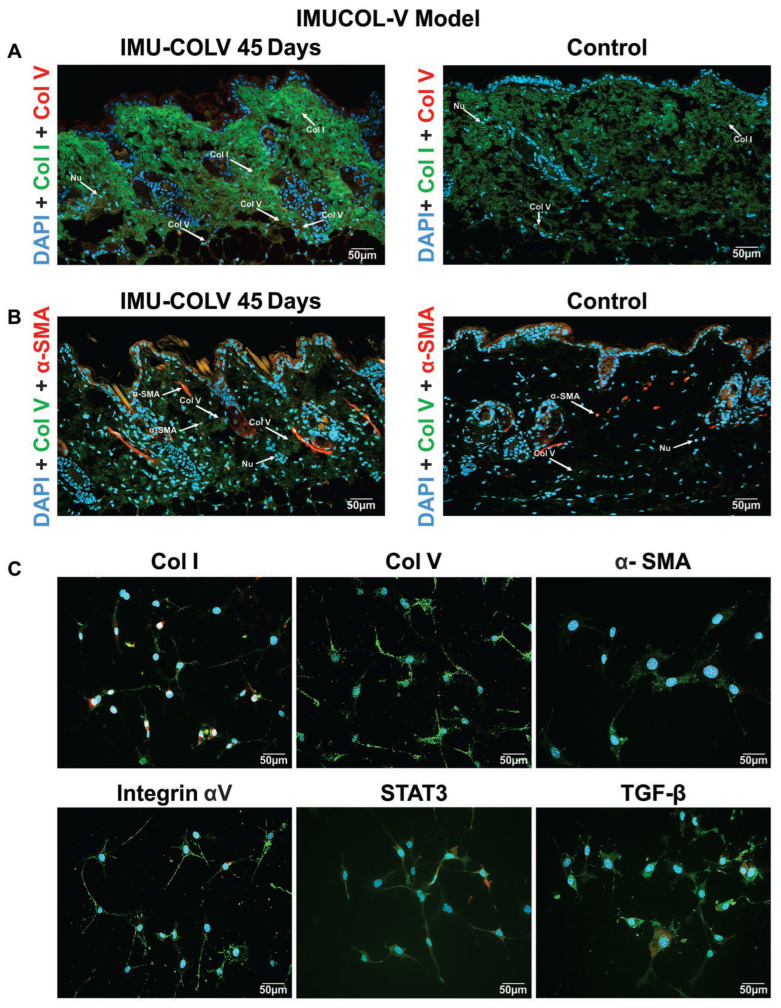
Immunofluorescence colocalization in skin tissue. (**A**) Representative images of colocalization of Col I (green) and Col V (red) immunostaining in the skin of mice from the IMU-COLV 45-day group and CT. (**B**) Colocalization of Col V (green) and α-SMA (red) immunostaining in the skin of mice from the IMU-COLV 45-day group and CT. The cell nuclei were stained blue with DAPI. (**C**) Skin fibroblasts from the IMU-COLV 45-day group. Immunofluorescence (green) shows the expression of Col I, Col III, Col V, α-SMA, Integrin αV, STAT3 and TGF-β in the cytoplasm of the fibroblasts. The nuclei of the cells are stained blue with DAPI. Nu: nucleus (original magnification: 400×).

By day 45, Col I and Col V fibers interacted to produce fibrillar disorganization and dermal matrix thickening. Col V fibers were more abundant than Col I fibers in the dermis of immunized mice (Figure 6A), consistent with Masson’s trichrome staining (Figure 2A). Col I fibers associated with Col V fibers to form thicker collagen structures—a pattern absent in CT mice (Figure 6A).

Myofibroblasts colocalized with Col V fibers, and immunolabeling revealed strong expression of integrin αV, TGF-β, and STAT3 within these cells (Figure 6B,C), indicating activation of pathways driving fibroblast differentiation and ECM deposition. Single-channel images for each marker and their corresponding merged composites are shown in Figure S3.

## 4. Discussion

In this study, we investigated the effects of Col V on fibroblasts and collagen remodeling in a mouse model, with emphasis on dermal structural changes and their relevance to fibrotic diseases such as SSc. Previous studies have explored the role of Col V in collagen structure and fibrotic responses; however, we uniquely provide an in vivo, longitudinal assessment of the interaction between Col V and Col I, as well as the activation of myofibroblasts, over an extended period (up to 45 days). These findings offer new insights into the role of Col V in collagen deposition and fibrotic matrix remodeling, which have been less thoroughly explored in prior research. Specifically, we observed fibrillar disorganization, collagen thickening, and long-term myofibroblast activation. Our results expand on previous findings by demonstrating that stimulation by Col V of Col I and Col III is dose-dependent, leading to both structural and quantitative collagen changes. A high proportion of Col V in the pericellular environment promotes activation of key signaling proteins, including integrin αV, TGF-β1, and STAT3. Taken together, these results suggest that Col V regulates both matrix composition and fibrotic signaling pathways, thereby promoting myofibroblast differentiation and collagen production. Although total protein expression of integrin αV, TGF-β, and STAT3 was increased, we did not directly evaluate their activation status (e.g., STAT3 phosphorylation or SMAD2/3 signaling). Therefore, our findings should be interpreted as indicative of a potential profibrotic signaling milieu rather than confirmed molecular activation.

Interestingly, we observed a non-linear response in α-SMA and Col I expression, with higher intensities detected at 25 µg/mL compared to 50 µg/mL. In contrast, increased Col V concentrations in fibroblast cultures promoted the expression of collagen types III and V. A higher proportion of Col V in the papillary dermis has previously been reported to be associated with the establishment of a niche of undifferentiated cells. These cells, in turn, synthesize substantial amounts of Col V, generating a positive feedback mechanism that sustains the integrity of this specialized microenvironment [25]. Conversely, in the reticular dermis, where Col V content is comparatively lower, undifferentiated cells are scarce, and reticular fibroblasts predominantly produce type I collagen while exhibiting elevated α-SMA expression [26]. Although this aspect was not specifically investigated in the present study, previous evidence has demonstrated that skin fibroblasts cultured in the presence of Col V—but not other collagen types—express markers characteristic of an undifferentiated phenotype, in addition to producing large quantities of Col V, reinforcing the pivotal role of Col V in maintaining dermal stem cell homeostasis [25]. Overall, our findings suggest that Col V contributes to the regulation and structural composition of the cutaneous extracellular matrix, although the underlying molecular mechanisms and signaling pathways remain to be elucidated.

Current treatment options for skin fibrosis and symptom prevention in SSc remain limited. In this study, we demonstrated that an increased proportion of Col V in the tissue matrix disrupts skin remodeling during the early stages of fibrosis, leading to abnormal fibrillogenesis and thereby partially explaining the clinical manifestations of skin fibrosis observed in SSc. Our results support the hypothesis that the composition and proportion of pericellular microenvironment play a critical role in fibrotic progression. These findings highlight Col V, together with its interaction with Col I, as a potential therapeutic target for managing skin fibrosis and mitigating the progression of SSc.

Given the established role of Col V in regulating collagen fibril assembly, the observed fibrillar disorganization and collagen thickening were expected, confirming the contribution of Col V to collagen network remodeling and fibril structure. Our study provides further insights into how these structural changes are linked to myofibroblast activation and the broader fibrotic response. In vivo, 45 days after immunization with Col V, mice developed a fibrotic skin phenotype characterized by increased collagen deposition, particularly of Col I and Col V, which resulted in skin thickening. This was accompanied by the appearance of myofibroblasts in the dermis, a feature associated with disease severity in SSc [27]. These findings align with the established role of increased collagen synthesis and myofibroblast accumulation in SSc-related skin fibrosis [28]. Thus, the altered fibrillary proportion induced by Col V reflects both matrix protein activation and the intrinsic biochemical properties of Col V.

The role of Col V in skin fibrosis is well documented, particularly its production by activated fibroblasts differentiating into myofibroblasts, a process regulated by vimentin and α-SMA [29,30]. In this study, we hypothesize that α-SMA is a key mediator of skin fibrosis progression in Col V-immunized mice. The timing of these events is critical for understanding the pathogenesis of SSc, as early-stage fibrosis may still be reversible with timely intervention. The capacity of Col V to modulate collagen fibrillogenesis—essential for the proper assembly of Col I and Col III fibrils—highlights its central role in fibrosis development [31,32,33]. Consistently, studies in Col V-deficient mice have demonstrated reduced fibrosis and less rigid collagen structures, further reinforcing its role in the disease process [34,35].

Furthermore, Col V immunization in our SSc model produced changes in collagen architecture and myofibroblast activation similar to those observed in the skin of patients with SSc. Previous clinical studies have also reported elevated Col V expression in early-stage SSc, distinguishing it from late-stage SSc [17]. These findings not only confirm the pathogenic role of Col V in fibrosis but also suggest it as a potential target for early therapeutic intervention. Our results therefore support the hypothesis that Col V plays a key role in the fibrotic processes of SSc, particularly in collagen deposition and remodeling. However, further studies, including direct comparative analyses with SSc skin samples, are needed to validate the relevance of our model to human disease.

The interplay between Col V, integrin αV, TGF-β, and STAT3 is a critical feature of fibrosis in our model. Integrin αV, which is upregulated in dermal fibroblasts from patients with SSc, facilitates the activation of latent TGF-β, thereby promoting fibroblast-to-myofibroblast differentiation and ECM deposition [36]. Recent studies have also shown that integrin αV activation during myocardial infarction stimulates myofibroblasts to produce large amounts of collagen, leading to fibrotic scar formation [37]. Consistent with these reports, our results confirmed this mechanistic pathway in the skin, with integrin αV enhancing TGF-β signaling and myofibroblast differentiation, thereby driving fibrosis. Although our study evaluated integrin α5 as a marker of fibroblast activation, we discuss the αV–TGF-β signaling axis as a plausible downstream pathway through which Col V may promote fibrosis, based on previous reports describing the activation of latent TGF-β by αV integrins [36,37]. Further studies are warranted to confirm the involvement of αV integrins in Col V–driven matrix remodeling. Elevated STAT3 expression, a key transcription factor in fibrosis, was also observed in our model and warrants further investigation. STAT3 is known to be upregulated in SSc and to contribute to fibroblast-to-myofibroblast differentiation and fibrosis progression. We did not assess the phosphorylated (active) form of STAT3; however, we acknowledge its importance in fibrosis. Future studies should examine STAT3 activation and its role in Col V-induced fibrosis, particularly in regulating myofibroblast activity and collagen production.

While our data demonstrate strong expression of Col V and integrin αV, TGF-β, and STAT3 in the dermis, these findings were obtained through high-resolution immunofluorescence analysis. Although additional quantitative approaches, such as qPCR or Western blotting, would further validate these results, our present focus was to define the spatial and structural context of pathway activation in situ. Future studies will explore these molecular interactions in greater depth, including analysis of STAT3 phosphorylation and downstream signaling targets. Furthermore, it will be of great value to assess the involvement of other pro-fibrotic signaling pathways, such as ERK/MEK, in Col V-induced fibroblast activation and ECM remodeling. Future experiments using selective MEK inhibitors (e.g., U0126 or PD98059) will be essential to confirm whether blockade of this pathway attenuates Col V–driven ECM induction and myofibroblast differentiation.

In conclusion, this study provides new insights into the role of Col V in collagen remodeling and fibrosis. Our findings demonstrate that Col V induces significant structural changes in the collagen matrix and activates myofibroblasts in a time-dependent manner. Further molecular investigations, including assessment of STAT3 activation and other fibrotic pathways, are needed to elucidate the underlying mechanisms. This study establishes a foundation for future research on the role of Col V in fibrotic diseases such as SSc. Furthermore, our findings highlight the importance of exploring alternative models that can expand the applicability of these findings while adhering to ethical guidelines.

## Data Availability

The original contributions presented in this study are included in the article/Supplementary Material. Further inquiries can be directed to the corresponding author.

## Supplementary Materials

The following supporting information can be downloaded at: https://www.mdpi.com/article/10.3390/cells14231865/s1, Figure S1: Negatives controls for immunofluorescence staining; Figure S2: Immunofluorescence colocalization in skin tissue; Figure S3: Negatives controls for Immunohistochemistry staining; Table S1: Culture features of myofibroblasts and collagens from healthy mice stimulated with Col V; Table S2: Histological, immunohistochemical and molecular markers parameters in the IMU-COLV model after 15, 30, and 45 days.

## Abbreviations

The following abbreviations are used in this manuscript:

SSc	Systemic Sclerosis
Col V	Type V Collagen
Col I	Type I Collagen
Col III	Type III Collagen
*Col5a1*	Collagen Type V Alpha 1 Chain (gene)
*Col3a1*	Collagen Type III Alpha 1 Chain (gene)
α-SMA	Alpha-Smooth Muscle Actin
ECM	Extracellular Matrix
IMU-COLV	Immunized with Collagen V
CT	Control
DMEM	Dulbecco’s Modified Eagle’s Medium
H&E	Hematoxylin and Eosin
DAPI	4′,6-Diamidino-2-Phenylindole
VEGF	Vascular Endothelial Growth Factor
RT-qPCR	Quantitative Reverse Transcription Polymerase Chain Reaction
BSA	Bovine Serum Albumin
STAT3	Signal Transducer and Activator of Transcription 3
TGF-β	Transforming Growth Factor Beta
