# Supplementary material for: Type V Collagen as a Critical Regulator of Fibrillar Matrix Remodeling in a Murine Model of Systemic Sclerosis
Source: Cells. 2025 Nov 26;14(23):1865. doi: 10.3390/cells14231865 (PMC12691064; doi:10.3390/cells14231865)
Supplement: Supplementary file 1 [file cells-14-01865-s001.zip › Supplementary Files/Supplementary figure legends.docx]

Figure S1: Negatives controls for immunofluorescence staining. Section incubated without primary antibody (only secondary antibody). (**a**) Anti-Goat 488 (green), (**b**) Anti-Mouse 488 (green), (**c**) Anti-Rabbit 488 (green), (**d**) Anti-Goat 546 (red), (**e**) Anti-Mouse 546 (red), (**f**) Anti-Rabbit 546 (red).

Figure S2: Negatives controls for Immunohistochemistry staining. Section incubated without primary antibody. (**a**) α-SMA, (**b**) VEGF.

Figure S3: Immunofluorescence colocalization in skin tissue. The nuclei were stained blue with DAPI. (**a**) Representative images of colocalization of Col I (green) and Col V (red) immunostaining in the skin of mice from the IMU-COLV 45-day group, DAPI/Col I/Col V (merge). (**b**) Representative images of colocalization of Col I (green) and Col V (red) immunostaining in the skin of mice from the CT, DAPI/Col I/Col V (merge). (**c**) Colocalization of Col V (green) and α-SMA (red) immunostaining in the skin of mice from the IMU-COLV 45-day group, DAPI/Col V/α-SMA (merge). (**d**) Colocalization of Col V (green) and α-SMA (red) immunostaining in the skin of mice from the CT, DAPI/Col V/ α-SMA (merge). (**e**) Negative Controls.
