# Supplementary material for: Type V Collagen as a Critical Regulator of Fibrillar Matrix Remodeling in a Murine Model of Systemic Sclerosis
Source: Cells. 2025 Nov 26;14(23):1865. doi: 10.3390/cells14231865 (PMC12691064; doi:10.3390/cells14231865)
Supplement: Supplementary file 1 [file cells-14-01865-s001.zip › Supplementary Files/Supplementary Table 1.docx]

**Table S1.** Culture features of myofibroblasts and collagens from healthy mice stimulated with Col V.

|  | Control | Stimulation with 25μg/mL | Stimulation with 50μg/mL |
| --- | --- | --- | --- |
| **After 24 hours** |  |  |  |
| Myofibroblast SMA+ | 668.3 ± 134.4 | 384.7 ± 64.21 | 528.0 ± 12.09 |
| Collagen type I | 526.4 ± 103.1 | 344.5 ± 52.52 | 432.7 ± 37.93 |
| Collagen type III | 614.0 ± 64.90 | 3262.0 ± 348.3* | 4941.0 ± 557.7^#^ |
| Collagen type V | 1716.0 ± 00.00 | 4279.0 ± 327.1* | 5562.0 ± 530.4^#^ |
| **After 48 hours** |  |  |  |
| Myofibroblast SMA+ | 394.4 ± 86.51 | 796.3 ± 85.01* | 464.1 ± 69.93 |
| Collagen type I | 321.8 ± 25.74 | 568.8 ± 55.89* | 370.0 ± 60.64 |
| Collagen type III | 501.3 ± 24.89 | 4330.0 ± 416.20* | 6903.0 ± 55.89^#^ |
| Collagen type V | 1887.0 ± 86.05 | 5804.0 ± 600.20* | 7315.0 ± 342.0^#^ |

**Notes:** *control vs stimulation with 25 μg/mL, P<0.05; ^#^control vs stimulation with 50 μg/mL, P<0.05. Values are the means (SEM), nine sites per well and three wells per condition were acquired, all values were computed in random, non-coincident fields per well.
