# Supplementary material for: Type V Collagen as a Critical Regulator of Fibrillar Matrix Remodeling in a Murine Model of Systemic Sclerosis
Source: Cells. 2025 Nov 26;14(23):1865. doi: 10.3390/cells14231865 (PMC12691064; doi:10.3390/cells14231865)
Supplement: Supplementary file 1 [file cells-14-01865-s001.zip › Supplementary Files/Supplementary Table 2.docx]

**Table S2.** Histological, immunohistochemical and molecular markers parameters in the IMU-COLV model after 15, 30 and 45 days (mean±SD).

|  | P | Effect size; IC95% | Control  15 days | IMU-COLV  15 days | Control  30 days | IMU-COLV  30 days | Control  45 days | IMU-COLV  45 days |
| --- | --- | --- | --- | --- | --- | --- | --- | --- |
| Thickness | <0.001 | 0.497  (0.35-0.68) | 159.4 ± 13.61^@,¢^ | 203.4 ± 39.43 | 173.0 ± 24.08^#^ | 224.1 ± 21.72^@^ | 190.5 ± 19.41 | 252.7 ± 299.16^¢,#^ |
| 4-Hydroxyproline | 0.0005 | 0.354 (0.20-0.58) | 6.04 ± 1.41^¢^ | 8.010 ± 1.46 | 6.423 ± 0.79 | 8.65 ± 2.42 | 5.39 ± 1.25^*^ | 8.908 ± 1.62^¢,*^ |
| VEGF | 0.0001 | 0.441 (0.25-0.68) | 52.32 ±11.82^¢^ | 57.15 ± 7.21^$^ | 53.54 ± 7.91^#^ | 63.21 ± 10.63 | 56.97 ± 6.66^*^ | 75.01 ± 4.47^¢,#,*,$^ |
| Collagen type I | <0.001 | 0.543 (0.35-1.00) | 12.40 ± 1.31^¢^ | 13.17 ± 1.63^&,$^ | 10.91 ± 2.09^#^ | 12.39 ± 1.95^%^ | 10.25 ±0.84^&,*^ | 15.50 ± 1.66^¢,#,*,$,%^ |
| Collagen type III | 0.32 | 0.018 (-0.04-0.32) | 10.62 ± 1.34 | 10.27 ± 1.160 | 9.09 ± 1.93 | 9.86 ± 1.330 | 9.66 ± 2.03 | 10.74 ± 1.33 |
| Collagen type V | <0.001 | 0.639 (0.475-1.00) | 8.44 ± 0.95^¢^ | 11.09 ± 2.13^&,$^ | 9.37 ± 1.22^#^ | 11.17 ± 2.19^§,%^ | 7.59 ± 1.25^&,§,*^ | 14.50 ± 2.08^¢,#,*,$,%^ |
| Myofibroblast  α-SMA | <0.001 | 0.466 (0.25-1.00) | 22.75 ± 7.18^@,¢^ | 35.21 ± 6.18 | 31.16 ± 15.20^®^ | 49.89± 12.46^@,®,§^ | 25.59± 12.36^§,*^ | 43.96 ± 9.53^¢,*^ |
| *Col3a1* | 0.65 | 0.095 (0.00-1.00) | 1.05 ± 0.35 | 1.28 ± 0.43 | 1.10 ± 0.50 | 1.48 ± 0.97 | 1.08 ± 0.46 | 1.23 ± 0.59 |
| *Col5a1* | 0.01 | 0.403 (0.08-1.00) | 0.82 ± 0.16^┼^ | 1.66 ± 0.66^┼,&^ | 0.90 ± 0.33 | 0.97 ± 0.34 | 0.90 ± 0.17^&^ | 1.15 ± 0.35 |

**Notes:** Values are the means (SEM) of 10 animals in each group. All values were computed in random, non-coincident fields per mice.

**Test used for comparison**:

ANOVA for: Collagen type I, Collagen type V, Myofibroblast α-SMA, *Col3a1*, *Col5a1*

Kruskal–Wallis for: Thickness, 4-Hydroxyproline, VEGF, Collagen type III

**Statistical difference (P<0.05) between:**

^┼^Control 15 days vs. IMU-COL V 15 days

^@^Control 15 days vs. IMU-COL V 30 days

^¢^Control 15 days vs. IMU-COL V 45 days

^®^Control 30 days vs. IMU-COL V 30 days

^#^Control 30 days vs. IMU-COL V 45 days

^&^Control 45 days vs. IMU-COL V 15 days

^§^Control 45 days vs. IMU-COL V 30 days

^*^Control 45 days vs. IMU-COL V 45 days

^$^IMU-COL V 15 days vs. IMU-COL V 45 days

^%^IMU-COL V 30 days vs. IMU-COL V 45 days
