# Supplementary figures and images for: Type V Collagen as a Critical Regulator of Fibrillar Matrix Remodeling in a Murine Model of Systemic Sclerosis
Source: Cells. 2025 Nov 26;14(23):1865. doi: 10.3390/cells14231865 (PMC12691064; doi:10.3390/cells14231865)

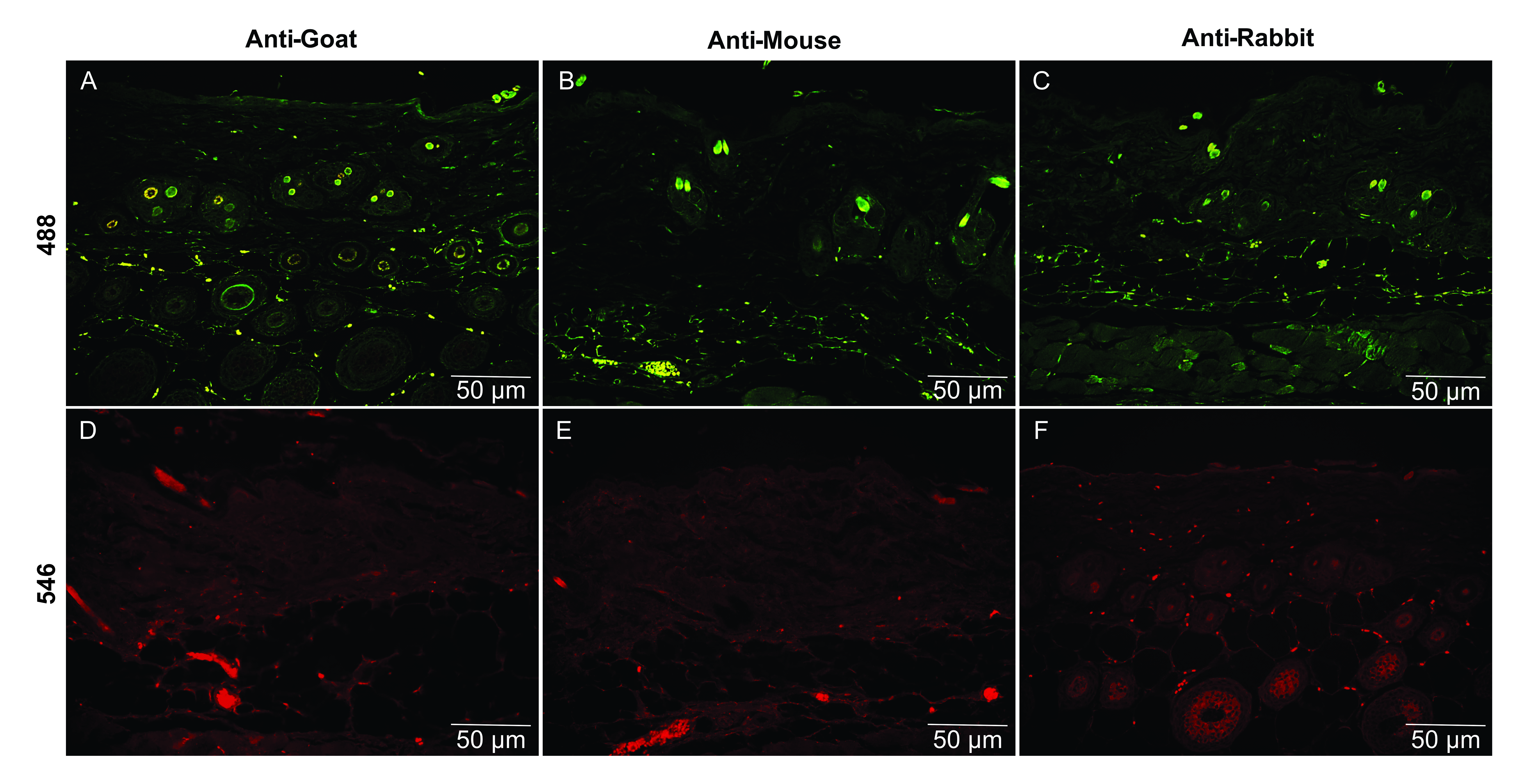

Supplement: Supplementary file 1 [file cells-14-01865-s001.zip › Supplementary Files/Figure S1- Negative Control IF JPEG.jpg]

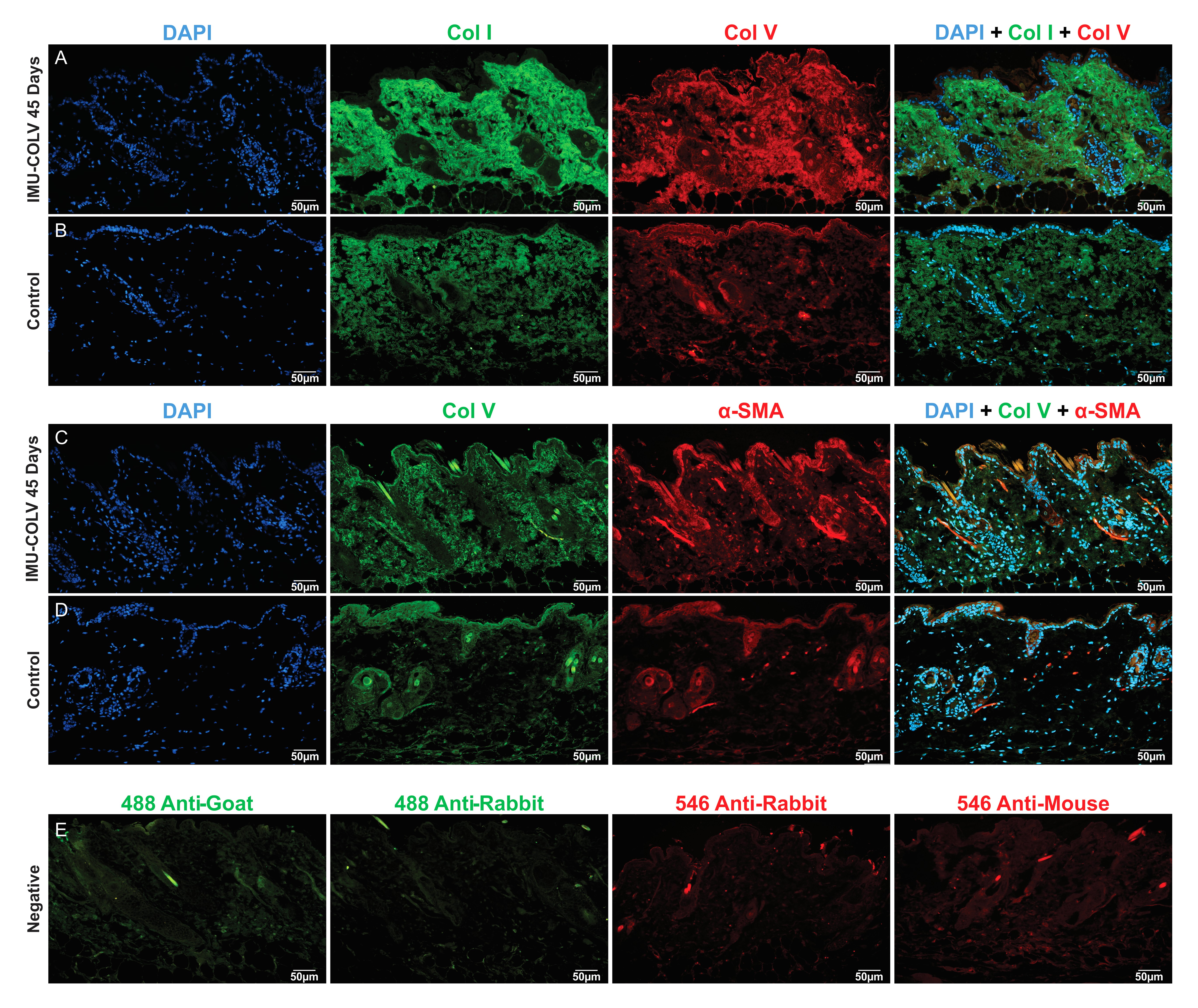

Supplement: Supplementary file 1 [file cells-14-01865-s001.zip › Supplementary Files/Figure S2-MERGE JPEG.jpg]

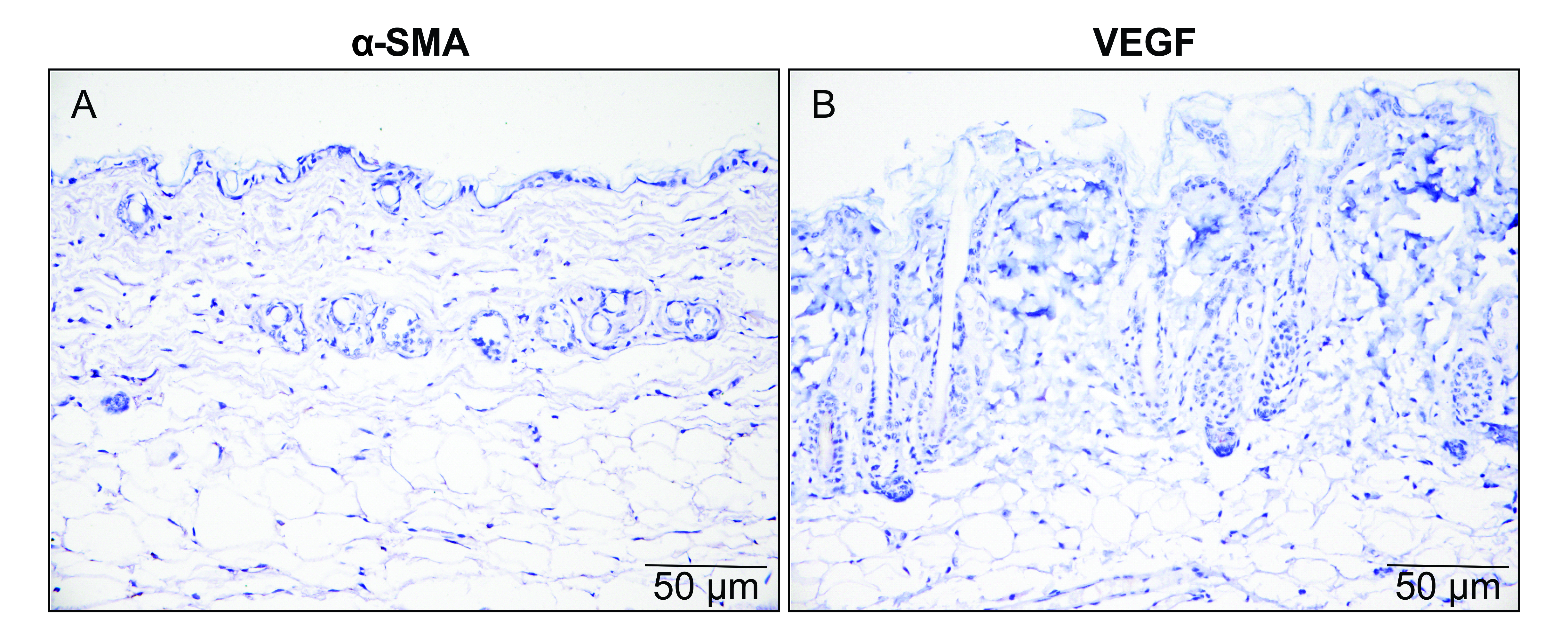

Supplement: Supplementary file 1 [file cells-14-01865-s001.zip › Supplementary Files/Figure S3- Negative Control IH JPEG.jpg]
